# RE: Low-Dose Aspirin in High-Risk Individuals With Screen-Detected Subsolid Lung Nodules: A Randomized Phase II Trial

**DOI:** 10.1093/jncics/pkab003

**Published:** 2021-01-23

**Authors:** Alain Braillon

**Affiliations:** Previously Senior Consultant, 80000 Amiens, France

I have concerns regarding the phase IIb trial (NCT02169271) of participants in lung cancer screening by helical low-dose computed tomography, which investigated the efficacy of low-dose aspirin on reduction in the size of subsolid lung nodules, viewed as potential adenocarcinoma precursors (1).

First, the prerequisites for conducting such a trial seem weak. On one hand, aspirin may effectively prevent the initial formation of tumors by impacting pathways fundamental to tumor initiation. On the other hand, aspirin may accelerate the development or spread of an in situ tumor—as suggested by the increase in cancer mortality in a population with a high prevalence of undiagnosed cancer in the ASPirin in Reducing Events in the Elderly (ASPREE) study. In ASPREE, low-dose aspirin was associated with a trend toward increased all-cause mortality driven by cancer deaths, including deaths from colorectal cancer (2). A smoking history of more than 20 packs per year ranks first among the causes of an increase in cancer mortality; we would appreciate additional information on participants’ age.

Second, as participants had a smoking history of more than 20 packs per year and two-thirds were current smokers, why were data about a smoking cessation program not reported? Smoking cessation is an important factor in clinical outcomes (cancer treatment effectiveness, overall survival, recurrence, risk of second primary malignancies, and quality of life). Could Bonanni and colleagues provide information about the percentage of current smokers who benefited from a proactive smoking cessation program (motivational interview and psychological support by trained professionals plus pharmacotherapy associating patches with oral “rescue”), formulations of nicotine (ie, sprays and lozenges) to suppress occasional cravings, “the belt and brace strategy” (3), and about the percentage of current smokers who quit at the end of the 1-year follow-up?

Cancer prevention must be comprehensive; screening, even with a costly sophisticated technique, is less effective if not included in a comprehensive program. Why are people who are attending cancer screening programs so rarely referred to multidisciplinary lifestyle interventions by trained professionals to treat major obvious risk factors (smoking, alcohol use, obesity, physical inactivity, etc)? There is robust evidence that lifestyle interventions by trained multiprofessional teams achieve sustained long-term beneficial effects (4). Indeed, tobacco, alcohol, and processed foods are industrial products causing “epidemics” of acute and chronic illness. Using this analogy, host reservoirs are industries; vectors for transmission include social marketing and other factors, such as societal controls exposure and availability as well as social determinants of health. Solutions require long-term policies (education, fair employment, universal healthcare, etc).

## Funding

None.

## Notes

**Role of the funder:** Not applicable.

**Disclosures:** None.

## Data Availability

Not applicable.

## References

[1] Bonanni B , Serrano D, Maisonneuve P, et al Low-dose aspirin in high-risk individuals with screen-detected subsolid lung nodules: a randomized phase-II trial. JNCI Cancer Spectrum. 2020;4(6). doi:10.1093/jncics/pkaa096 10.1093/jncics/pkaa096 PMC7771428 33409459

[2] McNeil JJ , Woods RL, Nelson MR, et al Effect of aspirin on all-cause mortality in the healthy elderly. N Engl J Med. 2018;379(16):1519–1528. 30221595 10.1056/NEJMoa1803955 PMC6433466

[3] Braillon A. Care for smoking cessation must be proactive and based on a combination of pharmacology and psychology. Oncologist. 2019;24(7):e607. 30890625 10.1634/theoncologist.2018-0817 PMC6656476

[4] Baumann S , Toft U, Aadahl M, Jørgensen T, Pisinger C. The long-term effect of a population-based life-style intervention on smoking and alcohol consumption. The Inter99 Study–a randomized controlled trial. Addiction. 2015;110(11):1853–1860. 26173928 10.1111/add.13052

